# Supplementary material for: JUN mRNA Translation Regulation is Mediated by Multiple 5’ UTR and Start Codon Features
Source: bioRxiv. 2023 Nov 17:2023.11.17.567602. Preprint. [Version 1] doi: 10.1101/2023.11.17.567602 (PMC10680820; doi:10.1101/2023.11.17.567602)
Supplement: Supplement 3 [file NIHPP2023.11.17.567602v1-supplement-3.pdf]

bioRxiv preprint doi: https://doi.org/10.1101/2023.11.17.567602; this version posted November 17, 2023. The copyright holder for this preprint (which was not certified by peer review) is the author/funder, who has granted bioRxiv a license to display the preprint in perpetuity. It is made available under aCC-BY 4.0 International license.

SUPPLEMENTARY MATERIALS

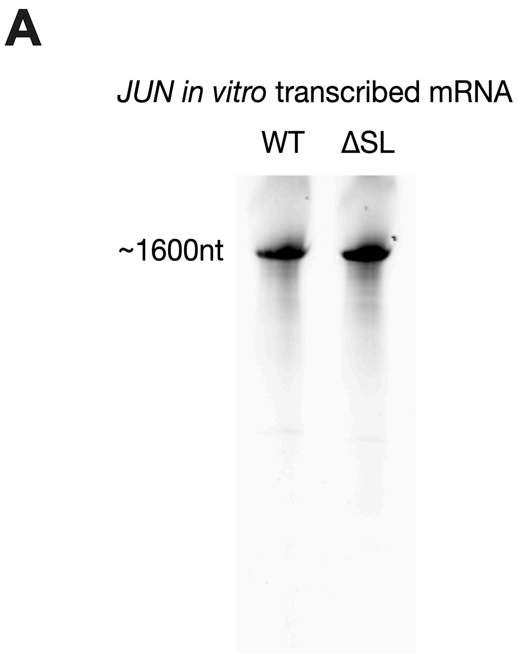

Supplementary Figure 1. Representative mRNA TBE-Urea gel.

6% TBE-Urea gel for *in vitro* transcribed mRNA for the WT or ΔSL *JUN* 5′ UTR and Nluc CDS reporter constructs. nt, nucleotide.

bioRxiv preprint doi: https://doi.org/10.1101/2023.11.17.567602; this version posted November 17, 2023. The copyright holder for this preprint (which was not certified by peer review) is the author/funder, who has granted bioRxiv a license to display the preprint in perpetuity. It is made available under aCC-BY 4.0 International license.

Supplementary Table 1. Conservation analysis for *JUN* uAUG and mAUG.

Compilation of species investigated for *JUN* AUGs conservation analysis, including the nucleotide and amino acid sequences of the 19-nucleotide *JUN* 5′ UTR and *JUN* CDS region that spans both AUG start codons and their translational context for each species, the corresponding Reference Sequence (RefSeq) accession numbers for each sequence, and the percent similarity of each sequence to the human *JUN* sequence.

Supplementary Table 2. Primers used in this study.

Compilation of primer sequences used for cloning and *in vitro* transcription amplification of each reporter construct used in this study.
